# Coupling Multi-Angle Light Scattering to Reverse-Phase Ultra-High-Pressure Chromatography (RP-UPLC-MALS) for the characterization monoclonal antibodies

**DOI:** 10.1038/s41598-019-51233-6

**Published:** 2019-10-18

**Authors:** Lorenzo Gentiluomo, Vanessa Schneider, Dierk Roessner, Wolfgang Frieß

**Affiliations:** 1 Wyatt Technology Europe GmbH, Hochstrasse 18, 56307 Dernbach, Germany; 2 Department of Pharmacy: Pharmaceutical Technology and Biopharmaceutics; Ludwig-Maximilians-Universitaet Muenchen, Butenandtstrasse 5, 81377 Munich, Germany

**Keywords:** Proteins, Analytical chemistry

## Abstract

Multi-angle light scattering coupled with size-exclusion chromatography (SEC-MALS) is a standard approach for protein characterization. Recently MALS detection has been coupled with ion-exchange chromatography (IEX) which demonstrated the feasibility and high value of MALS in combination with non-sized-based fractionation methods. In this study we coupled reverse-phase ultra-high pressure liquid chromatography (RP-UPLC) with a low-dispersion MALS detector for the characterization of intact monoclonal antibody (mAbs) and their fragments. We confirmed a constant refractive index increment value for mAbs in RP gradients, in good agreement with the values in literature for other classes of proteins. We showed that the impurities eluting from a RP column can often be related to aggregated species and we confirmed that in most cases those oligomers are present also in SEC-MALS. Yet, in few cases small aggregates fractions in RP-UPLC are an artifact. In fact, proteins presenting thermal and physical stability not suitable for the harsh condition applied during the RP separation of mAbs (i.e. organic solvents at high temperature) can aggregate. Further, we applied RP-UPLC-MALS during a long term stability studies. The different principle of separation used in RP-UPLC- MALS provides an additional critical level of protein characterization compared to SEC-MALS and IEX-MALS.

## Introduction

Light scattering is one of the widely-used techniques for the characterization of macromolecules and particles in solution in biological and biopharmaceutical sciences^1^. By far the most common application of light scattering in this field is the determination of mass and size of proteins by means of multi-angle light scattering coupled to size-exclusion chromatography (SEC-MALS)^2^ or field flow fractionation (FFF-MALS)^3^. Other important applications include the characterization of protein conformational and colloidal stability and the characterization of both specific and non-specific protein-protein interaction^1^. The use of MALS with fractionated samples yields a calculation of the absolute molecular weight (M_w_) at each point of the chromatogram. As the M_w_ estimated by the retention time is often inaccurate^4,5^, SEC-MALS provides a useful tool for determination of accurate monomer and fragment M_w_, oligomeric state and hydrodynamic radius (R_h_)^1,2,6^. Recently the advantages of coupling MALS with ion exchange chromatography (IEX) have been demonstrated^7^. IEX separates proteins according to surface charge based on differences in ionic interaction with the support matrix^8^. The different principle used in the separation of IEX-MALS provides additional critical information and can resolve SEC-MALS shortcomings^7^. In this study, we coupled MALS with another type of liquid chromatography, reversed-phase (RPLC). RPLC is a highly promising technique to study chemical changes^9–11^ and to quantify^12,13^ peptides and proteins, including monoclonal antibodies (mAbs). Historically, the use of RP to monitor intact mAb was limited because the complex hydrophobic and hydrophilic nature of these large proteins caused poor recovery and limited resolution. More recently, the use of columns with large pores (300 $\dot{\text{A}}$) at high temperatures (60–75 °C) in combination with non-traditional solvent system containing ion pairing agents has been consolidated as standard procedure for the analysis of mAbs, overcoming previous difficulties^14,15^. Small chemical differences cannot be separated by standard RP-HPLC^16^, as they are often insufficient to yield significant changes in polarity^17^. Here, we took advantage of ultra-high pressure LC (UPLC) instrumentation to further refine the separation of mAb species and their derivatives. We investigated RP-UPLP-MALS for mAb characterization, focusing on two common applications: (i) analysis and characterization of mAb fragments, which are typically studied by mass spectrometry, (ii) analysis of mAbs after long term storage. The former is a real-time stability testing which permits the establishment of recommended storage condition and shelf life of the bio-therapeutic products. The addition of MALS allows the M_w_ assignment for each individual peak in the chromatogram enabling differentiation between chemical variants of the monomeric form and other impurities or degradation products as aggregates and fragments.

## Results and Discussion

### RP-HPLC-MALS technique

**Figure 1:**
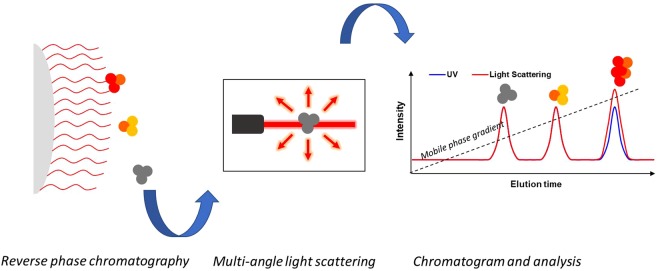
Schematic illustration of the RP-UPLC-MALS method. A protein sample is injected in the RP chromatography column in-line with a MALS detector. The protein interacts with the hydrophobic matrix.

The principle of RP-HPLC-MALS is the combination of RP chromatography with an online MALS detector. As shown in Fig. 1, multiple hydrophobic areas of protein molecules interact with the alkyl silane-derivated surface of the stationary phase. The separation is achieved by decreasing the water concentration in the mobile phase increasing the organic solvent fraction (e.g. acetonitrile). This in turn weakens the hydrophobic attraction of the protein to the column. During elution from the column the molecules are then introduced into a concentration detector (i.e. UV) and subsequently in a MALS detector. Using these detectors to measure the M_w_ of eluting molecules is especially important as no column calibration procedure, analogous to that of analytical SEC, can be applied to relate the size of a molecule to its hydrophobic interaction with a column matrix.

### Development of RP-UPLC-MALS

Good RP-HPLC conditions for intact protein analysis are typically achieved with a UPLC, a stationary phase with short alkyl chain length and large pore size, a strong ion-pairing agent and an adequate gradient decreasing the water content of the mobile phase at high temperature^9^. We coupled a low-volume, low-dispersion MALS detector to our UPLC system allowing for small peak width and high resolution. Six different IgG1s (PPI01, PPI02, PPI03, PPI04, PPI10, PPI13), one IgG2 (PPI17), one bispecific (PPI08), and one protein-drug conjugate (PPI18) were used to develop and assess our RP-UPLC-MALS method. IFNα2a served as a reference, as RPLC is a well-established technique to detect its chemically-changed species^18–21^. During the development of the RP-UPLC-MALS method column type, temperature, flow rate, injection volume, mobile phase and gradient were evaluated^14,15^. Some proteins presented better resolution with the BEH-300 C4 column compared with the Zorbax 300SB-C8 column. However, we noticed a fast decrease of efficiency with the BEH-300 C4 after just 400 injections, while the Zorbax 300SB-C8 showed good robustness. This is possibly due to the fact that the C4 phase chemistry is less resistant to hydrolysis in acidic media than the C8 phase chemistry. As screen of proteins in multiple formulation and across many time points involve thousands of injections, we selected the Zorbax 300SB-C8 as workhorse.

In order to determine M_w_ correctly, it is necessary to know the refractive index increment of solute in solution value dn/dc and the concentration for each slice of a peak. It has been shown that MALS is compatible with RP elution gradients^22^. Different classes of proteins have been investigated in literature with various mobile phase compositions containing aqueous buffer and acetonitrile yielding a dn/dc values close to 0.175 ml/g^22–25^. It has been shown that assuming a constant dn/dc in the narrow interval of an eluting peak only induces an error at most 3–4%^22^. This is due to the fact that the solvent refractive index changes only very slightly within the time frame of peak elution^26^. We first calculated the protein M_w_ using the dn/dc of proteins in water at 660 nm of 0.185 mL/g^27^. The obtained M_w_ was approx. 25% below the M_w_ calculated based on the primary sequence. Consequently, we fixed the M_w_ of the monomer as calculated from the primary sequence and confirmed by SEC-MALS to obtain a dn/dc in the RP-MALS eluent. This yielded a dn/dc value of 0.1742 +/− 0.0017 mL/g for the proteins, which is in very good agreement with the literature^22–25^, and was used for calculating the M_w_ of the investigated proteins.

### Analysis of intact monoclonal antibodies using RP-UPLC-MALS

**Figure 2:**
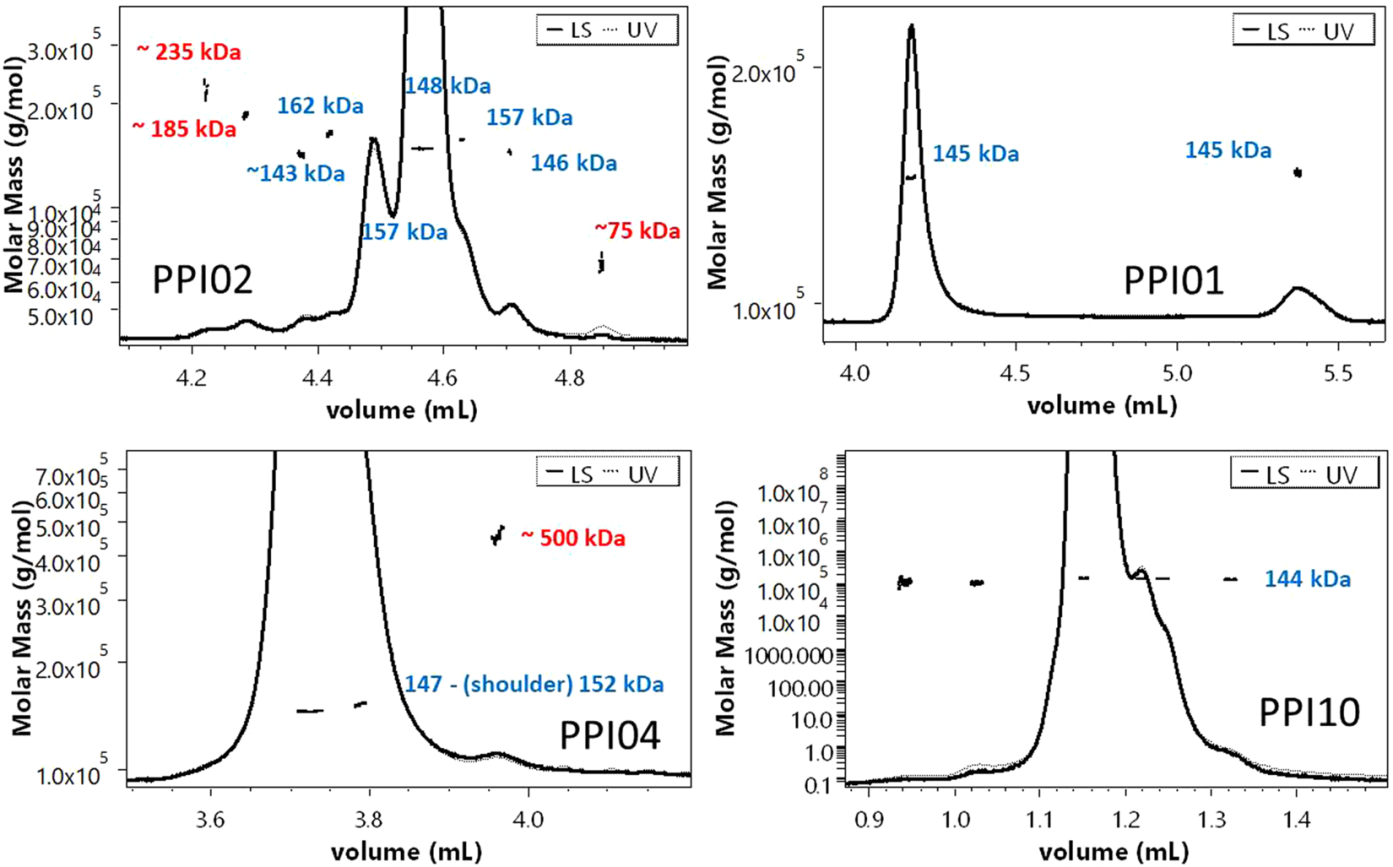
RP-UPLC-MALS of mAbs. Typical chromatograms showing the UV and the MALS signals of PPI02, PPI01, PPI04 and PPI10 analyzed by RP-UPLC-MALS. The MW of the monomer, aggregates/fragments, and dimers are highlighted in blue and respectively. (*) denotes aggregates.

**Figure 3:**
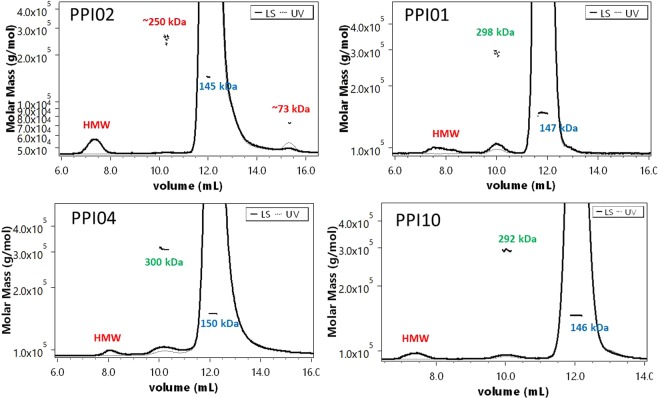
SEC-MALS of mAbs. Typical chromatograms of the proteins investigated by SEC-MALS showing UV and LS signals along superimposed with calculated molar mass. The M_W_ of the monomer, aggregates/fragments, and dimers are highlighted in blue, red and green, respectively. HMW stands for high-molecular weight species, which are usually not separated, and in all our investigated cases presented no UV detectable signal. (*) denotes aggregates; (**) denotes dimers.

Proteins with similar size cannot be separated by SEC, but if they have a different hydrophobicity they can be separated by RP-UPLC. In our study we encountered three cases: (i) The M_w_ of all the peaks reflects monomeric variants (e.g. PPI01 and PPI10), (ii) The main peak represents a monomeric form while other impurity peaks are identified as aggregates (e.g. PPI04), (iii) The main peak represents a monomeric form while other impurities peaks are either identified as aggregates, fragments or close to, but not equal within the experimental error, to the monomer M_w_ (e.g. PPI02) (Fig. 2). Dimers detected in SEC-MALS (Fig. 3) were not found in RP-UPLC-MALS (Fig. 2). As the RP-UPLC recovery was often close or exactly 100% (Table 1) we hypothesize that (i) the monomer-dimer equilibrium is shifted completely towards the monomeric form in the RPLC eluent, (ii) the dimers are prompted to further aggregation, (iii) the dimers are lost over the column. Both RP-UPLC-MALS and SEC-MALS confirmed the absence of oligomers beyond the dimers visible in SEC for PPI01 and PPI10 (Fig. 2). Similar conclusions were reached for PPI13, PPI08 and PPI17 (Supplementary information (SI) 1). Differently, PPI04 (Fig. 2) and PPI18 (SI 2) showed a very small fraction of oligomers by RP-UPLC-MALS, which were not detected in SEC-MALS (Fig. 3).These oligomers may have been induced by the high temperature of 75 °C applied during the RP separation. The first temperature of unfolding (T_m1_), the temperature of aggregation (T_agg_), and the diffusion interaction parameter (k_D_) for PPI01, PPI02, PPI03, PP10 and PP17 are 66, 61 °C and 5.6 mg/L (data averaged from 24 formulation conditions, Gentiluomo L, *et al*.)^28^ as compared to 54 °C, 47 °C and 4.7 mg/L resp. for PPI18 and 64 °C, 55 °C and −1.9 mg/L for PPI04. This lower thermal and/or colloidal stability of PPI18 and PP4 could explain their susceptibility to aggregation under the RP conditions. Finally, PPI02 showed aggregates and fragments (highlighted in red in Fig. 2) that were also detected in SEC-MALS (Fig. 3). The averaged M_w_ of the PPI02 aggregates from SEC-MALS and RP-UPLC-MALS are respectively of 250 kDa and 235 kDa. This difference is probably due to the high error in the M_w_ calculations, which is in turn due to the small concentration of such aggregates. Further, the 235 kDa aggregate in RP-UPLC-MALS is not baseline separated. PPI02 further presented a series of peaks and shoulders with 5 to 15 kDa difference to the monomer M_w_, which were not visible by SEC-MALS. The M_w_ difference may be possibly due to post-translational modifications of the IgG. These typically include methionine oxidation, asparagine and glutamine deamidation, N-terminal acetylation or cyclization, glycation of lysine and variable glycosylation^29^. Physically, the refractive index increment is insensitive to the long-range structure of macromolecules^27^ and is nearly independent of its amino acid composition^30^. However, carbohydrate moieties do affect the refractive index value^31^. This would suggest that PPI02 comes with a high degree of variation in glycosylation.

**Table 1:** Information on the investigated protein. The theoretical M_w_ is calculated from the primary sequence. Mass recovery is calculated over all the visible UV peaks as described in material and method.

Type	Provider	Ɛ at 280 nm (mg/ml/cm)	Theoretical M_W_ (kDa)	MALS M_w_ (kDa)	Mass recovery	pI	Notes	ID
IgG1λ	AstraZeneca	1.56	144.8	144.1 ± 0.2%	99.9%	7.96	—	PPI01
Human IgG1κ	AstraZeneca	1.47	148.2	148.1 ± 0.1%	100%	8.53	—	PPI02
Human IgG1κ	AstraZeneca	1.435	144.8	144.6 ± 0.2%	100%	8.44	WT IgG	PPI03
IgG1λ YTE	AstraZeneca	1.755	146.2	146.5 ± 0.1%	97.2%	8.99	—	PPI04
IgG1κ + scFv	AstraZeneca	1.57	204.4	204.4 ± 0.1%	98%	9.2	Bispecific	PPI08
Human IgG1	AstraZeneca	1.533	144.2	144.6 ± 0.2%	96.5%	8.95	—	PPI10
Human IgG1κ	AstraZeneca	1.66	148.9	148.7 ± 0.2%	100%	9.04	—	PPI13
IgG2κ	AstraZeneca	1.31	145.1	145.6 ± 0.3%	99.9%	7.78		PPI17
HSA-NEP	AstraZeneca	1.04	146.7	146.3 ± 0.1%	100%	5.8	Conjugate	PPI18
Intα-2A	Roche	0.972	19.2	20.1 ± 7.5%	100%	5.97	—	PPI30

### Characterization of Fab and Fc fragments

**Figure 4:**
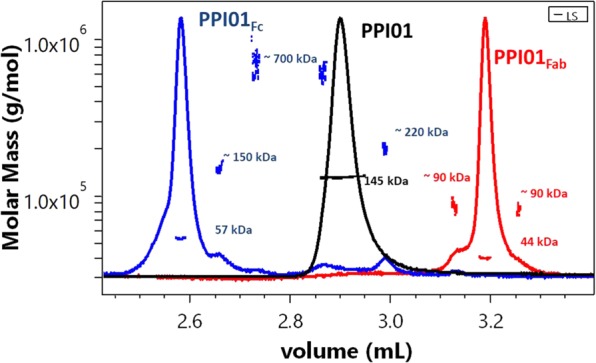
RP-UPLC-MALS of PPI01 and its fragments. PPI01 Fc fragment, PPI01 (whole mAb) and PPI01 Fab fragment are plotted in blue, black and red lines, respectively.

Complete proteolytic digestion of mAb (peptide mapping) followed by RP-UPLC coupled with mass spectrometry (MS) is a well-established method for the identification and quantification of chemical modification of mAbs^32,33^. Alternatively, the analysis by MALS of large fragments, such as Fab and Fc, requires little sample preparation and can provide a high-throughput alternative. The preparation and purification of the fragments was performed as described in material and methods. Subsequently, we investigated the Fab and Fc fragments of PPI01 by RP-UPLC-MALS. The Fc fragment eluted before the intact mAb which in turn eluted before the Fab fragment (Fig. 4). The latter exhibited two shoulders on the left and right of the 47 kDa monomer with a M_w_ close to that of a Fab dimer (~90 kDa). The Fc fragment elutes with a series of peaks after the main peak of ~110, ~700, ~170 kDa with longer elution time. SEC-MALS measurements on the purified fragments confirmed the presence of Fab dimer and of Fc dimer and trimer (fragments showed in SI 3, intact mAb showed in Fig. 3). However, the 700 kDa Fc aggregate was not detected in SEC-MALS. As mentioned previously, the formation of small fraction of high molecular-weight oligomers due to the RP conditions can affect proteins with insufficient thermal and/or colloidal stability. PP01 shows averaged T_m1_, typically reflecting unfolding of the CH2 domain and T_m2_, typically reflecting unfolding of the CH3 and Fab fragment, of 64 and 77 °C^34–36^. This would explain the higher susceptibility of the Fc fragment to unfolding and aggregation. Thus, it could be useful to couple MALS with RP-UPLC-MS to differentiate between monomer and aggregates peak before analyzing the MS spectra.

### Long term stability studies

**Figure 5:**
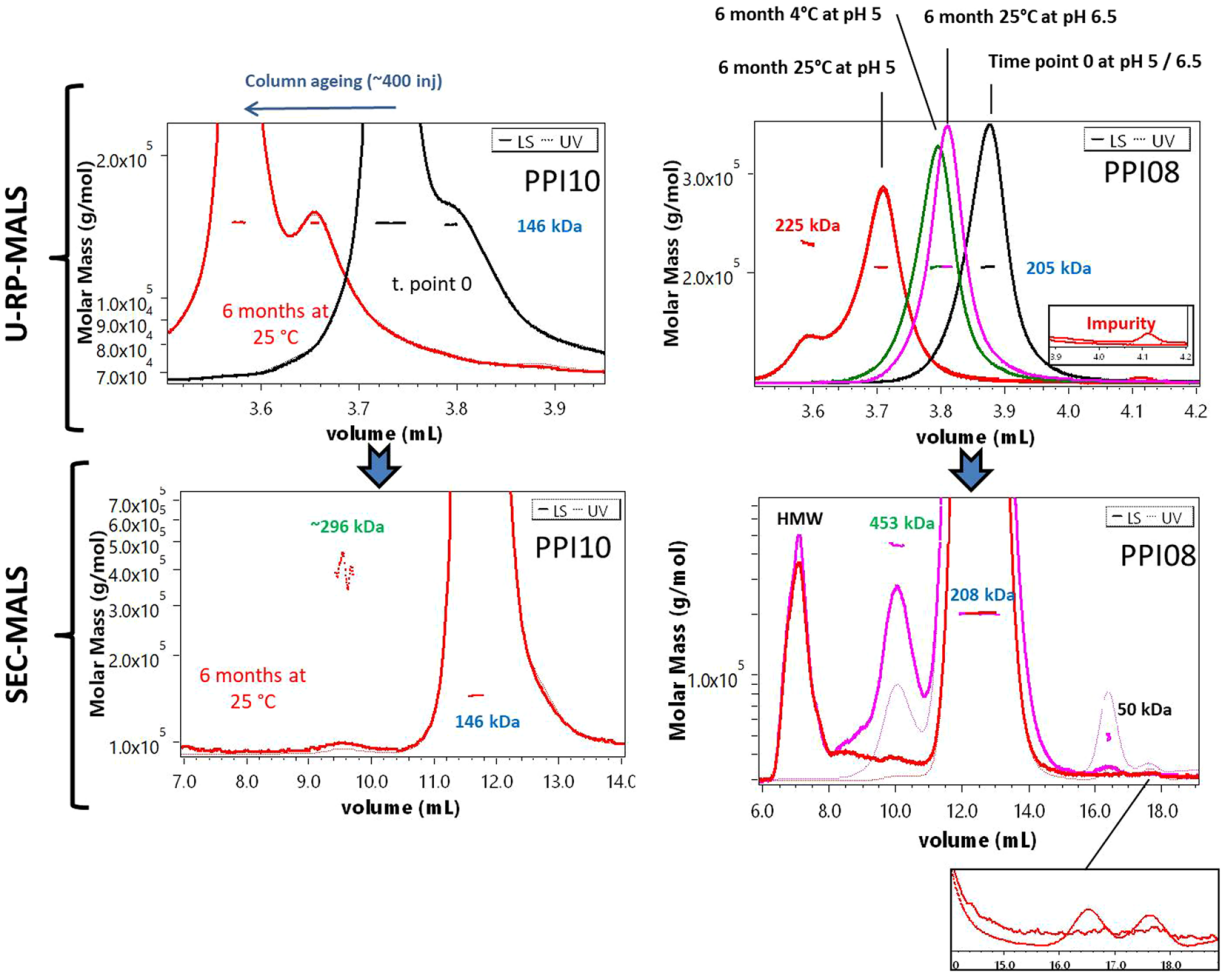
RP-UPLC-MALS and SEC-MALS of mAbs for long term stability studies. Typical chromatograms of the long-term-storage study, showing the regions of eluting sample. Top: RP-UPLC-MALS chromatograms; bottom: SEC-MALS chromatograms. Left: PPI10; right: PPI08. The M_w_ of the monomer, aggregates/fragments, and dimers are highlighted in blue, red and green, respectively. HMW stands for high-molecular-weight species, which are usually not separated, and in all our investigated cases presented no detectable UV signal. A magnified section shows the impurities for PP08. The shifting of the chromatograms at different time points is due to column ageing. PPI10 is shown in one formulation only (His 10 mM at pH 6.5), where the chromatograms before and after 6 months at 25 °C are depicted in black and red, respectively. PPI08 is shown formulated at pH 6.5 (His 10 mM) before stress, in black, and after 6 months at 25 °C, in magenta, and formulated at pH 5 (His 10 mM) before, in black, and after 6 months at 4 °C, in green, and 6 months at 25 °C, in red. PPI08 fragments are zoomed.

Finally, we performed a long term stability study and analyzed samples with the RP-UPLC-MALS method developed herein to learn whether we can gain additional insights from the MALS information on the chemical stability of our proteins. PP02, PP03, PP04, PP08, PP10, PP13 were tested in 8 different formulations for six month at 4 °C and 25 °C (see SI 4 for the formulations list). We observed an overall high chemical stability. Significant changes upon storage stress occurred only in a few conditions. PP10, formulated in 10 mM His at pH 6.5 stored at 25 °C, exhibited an increased hydrophobicity of the shoulder, presenting the same M_w_ of the monomer (Fig. 5). Chemical changes can perturb the local conformation backbone of proteins, such in the case of deamidation, the most common hydrolytic reaction for protein, and Asp isomerization. Conformational variants of proteins often present increased hydrophobicity and are more prone to aggregate^37^. Other chemical reactions, such Met oxidation, could on the other side decrease the hydrophobicity of proteins^38^. However, RP-UPLC-MALS cannot provide mechanistic insight behind an increased hydrophobicity after isothermal stress. For such purpose mass spectroscopy, which could be coupled with RP-UPLC-MALS, could provide quantitation of degradation products, such in the case of deamidation products^39^. PPI08 stored at 25 °C in 10 mM Histidine at pH 5 showed a new peak with an M_w_ of 225 kDa, which was not observed in any other formulation and was not noticeable in SEC-MALS (Fig. 5). This aggregate is probably formed by a mixture of fragments formed during the stress e.g. Fab, Fc, Heavy chain or by a complex formed by monomer and light chain. Comparison with SEC-MALS, confirmed the presence of fragments (Fig. 5). As baseline separation was not obtained between the monomer and the dimer, we could not tell whether the small complex is present in the formulation or formed during the RP separation. Regardless, MALS provided the exact M_w_ of the peaks eluting upon RP-UPLC, which allowed differentiation between chemical variants of the monomer (i.e. in cases of PPI10) and aggregates (i.e. in case of PPI08) formed during long term storage.

## Conclusions

We successfully coupled RP-UPLC with MALS to calculate the M_w_ of each eluting peak of intact mAbs and of Fc and Fab fragments. The different principle of separation used in RP-UPLC-MALS provides an additional critical level of protein characterization compared to SEC-MALS and IEX-MALS. RP is one of the most promising analytical techniques to analyze proteins^11,12,40^. Yet, peaks eluting from the column can often be related to aggregated species. Thanks to MALS, it is possible to tell whether an impurity is indeed a chemical variant of the monomer, an aggregate or a fragment. Furthermore, we highlight that the organic solvent and the temperature applied during the RP separation of mAbs could artificially induce aggregates which may lead to false interpretation of protein purity. Nonetheless, MALS could not be enough to describe detailed mechanisms and further coupling with MS (i.e. RP-UPLC-MALS-MS) could prove in the future natural development to characterize RP chromatograms.

## Material and Methods

### Sample preparation

Five antibodies IgG1s (PPI02, PPI03, PPI04, PPI10, PPI13), one bispecific antibody (PPI08), one IgG2 (PPI17), and one HSA-fusion protein (PPI18) were provided by AstraZeneca (Cambridge, UK). Interferon alpha-2a (PPI30) was provided from Roche Diagnostics GmbH. A summary of the protein’s physical properties is listed in Table 1. The proteins were dialyzed overnight using Slide-A-Lyzer™ cassettes (Thermo Fisher Scientific, Waltham, USA) with suitable membrane cut-off against excess of 10 mM of histidine HCl buffer with pH 5.0, 5.5, 6.0, 6.5, 7.0, 7.5. The excipient (e.g. NaCl) stock solutions were prepared in the respective buffers. Protein concentration was measured on a Nanodrop 2000 (Thermo Fisher Scientific, Waltham, USA) using the protein extinction coefficient calculated from the primary sequence. All conditions were prepared in 1.5 mL non-coated PP Eppendorf tubes. Finally, the formulations were sterile-filtered with 0.22 μm cellulose acetate filters from VWR International (Darmstadt, Germany). The purity of the proteins was studied by SEC and cEIF (SI 5).

### Ultra-high-pressure reverse-phase chromatography combined with multi-angle light scattering (RP-UPLC-MALS)

RP-UPLC-MALS was conducted on an ACQUITY UPLC H-Class system (Waters, UK) equipped with a quaternary pump, an autosampler, UV detector and a μDAWN detector (Wyatt Technology, USA). The separation was performed with both an Acquity BEH-300 C4 (Waters, UK) and a Zorbax 300SB-C8 column (Agilent Technologies, USA). The samples were diluted to 1 mg/mL before injection. For monoclonal antibodies a pilot gradient of 20 to 40% of eluent B in A over 20 minutes was used. Eluent A consisted of 10% w/v acetonitrile and 0.1% w/v trifluoracetic acid in ultrapure water. Eluent B consisted of 0.1% w/v trifluoracetic acid in acetonitrile. The flow rate was 0.2 mL/min. The column oven temperature was set at 75 °C. A preheater was included before the column. Subsequently, depending on the protein and the column used the gradient was fine-tuned. All methods were based on a gradient from 20–25 to 40%. On-column adsorption of the mAbs was evaluated systematically and almost complete mass recovery was reached for all the protein (Table 1). All the calculations were performed with ASTRA V7.1 software (Wyatt Technology, USA). Mass recovery is calculated from the injected mass versus the calculated mass from the concentration detector (i.e. UV). Therefore, to achieve an accurate determination of the mass recovery the sample concentration needs to be measured accurately. Thus, the concentration was measured again before injection in real triplicates by a Nanodrop One (Thermo Fisher Scientific, Waltham, USA). The theoretical extinction coefficients were double-checked re-calculating the values from the RI monomeric peaks during the SEC-MALS experiments. PPI30 (int-2alpha) was used as a standard. Finally, to achieve a flat baseline, we collected and subtracted the blanks by the algorithm included in the ASTRA V7.1 software.

### Size-exclusion chromatography combined with multi angle light scattering (SEC–MALS)

SEC-MALS was conducted on Agilent 1260 Bio-Inert system with a variable wavelength UV detector operated at 280 nm (Thermo Fischer Scientific, USA), followed by a TREOS II detector (Wyatt Technology, USA) and an Optilab T-rEX (Wyatt Technology, USA). The temperature controlled-autosampler was kept at 4 °C. Separation was performed with a Superdex 200 increased 10/30 GL column. Data were collected and processed using the ASTRA® software V7.2 (Wyatt Technology, USA). The aqueous mobile phase consisted of 38 mM NaH2PO4, 12 mM Na2HPO4, 150 mM NaCl and 200 ppm NaN3 at pH 7.4 dissolved in HPLC-grade water, filtered through Durapore VVPP 0.1 m membrane filters (Millipore Corporation, USA). The samples were centrifuged and injected in duplicates of 25 µl.

### Stress assay

0.2 mL of each protein solution was aliquoted at a concentration of 1 mg/mL and filtered in 0.5 mL sterile non-coated PP Eppendorf tubes. The samples were incubated at 4 °C and 25 °C, for 6 months. After storage, the samples were quenched in an ice bath, left at 4 °C and measured within two weeks. Sample concentration was measured after the stress in real triplicates by a Nanodrop One (Thermo Fisher Scientific, Waltham, USA). Similarly, the pH was measured after the stress showing no changes within the experimental error (i.e. ±0.1).

### Preparation and purification of Fab and Fc Fragments

Immobilized Papain (Thermo Fisher Scientific, Waltham, USA) was used to digest PPI01 into its Fab and Fc fragments. PPI01 at 20 mg/mL was pipetted into 15 mL glass vial, the vial capped with the resin separator provided with the kit to remove all the air-liquid interface. The vial was gently rotated by a Sunlab rotator SU1100 for 5 h at 37 °C. An ÄKTA purifier 10 (GE Healthcare, Uppsala, Sweden) equipped with a Pierce Protein A chromatography cartridge (Thermo Fisher Scientific, Waltham, USA) (column volume, CV = 5 ml) was used to separate Fc (and undigested mAb) from the Fab fragments. The binding buffer was composed of 100 mM sodium phosphate with 150 mM NaCl at pH 7.2. The column was equilibrated with 2 CV of binding buffer with a flow of 2 ml/min. Fractions were collected in 15-ml PP tubes using a Frac 920 fraction collector (GE Healthcare, Uppsala, Sweden) capturing any unbound species e.g. Fab. The elution buffer (100 mM sodium phospate at pH 3) was kept at 100% over 7 CV. The eluting protein was collected in 15-ml PP tubes using the fraction collector, and was immediately neutralized with a 1 M sodium phosphate buffer at pH 8.5. Ultrafiltration was performed using Vivaspin® tubes with a 10 kDa MWCO PES membrane (Sartorius Stedim Biotech, Göttingen, Germany). Success of the purification was monitored by HP-SEC (see 3.4).

## Supplementary information

Supplementary

## Data Availability

The raw data presented in this work will be made available via a specially-designed publicly-available database (https://pippi-data.kemi.dtu.dk/), currently in phase alpha.
