# Supplementary material for: Coupling Multi-Angle Light Scattering to Reverse-Phase Ultra-High-Pressure Chromatography (RP-UPLC-MALS) for the characterization monoclonal antibodies
Source: Sci Rep. 2019 Oct 18;9:14965. doi: 10.1038/s41598-019-51233-6 (PMC6800455; doi:10.1038/s41598-019-51233-6)
Supplement: Supplementary file 1 — Supplementary [file 41598_2019_51233_MOESM1_ESM.pdf]

Supplementary information for

Coupling Multi-Angle Light Scattering to Reverse-Phase Ultra-High-Pressure Chromatography (RP-UPLC-MALS) for the characterization of monoclonal antibody

Lorenzo Gentiluomo, Vanessa Schneider, Dierk Roessner, Wolfgang Frieß

The file includes:

SI 1. PPI-8, PPI-13 & PPI-17 RP-UPLC-MALS Chromatograms
SI 2. PPI-18 RP-UPLC-MALS Chromatogram
SI 3. Fab & Fc SEC-MALS Chromatograms
SI 4. Formulations list for long term stability studies
SI 5. Purity data (i.e. cIEF and SEC) of the protein bulk

Supplementary information

SI 1. RP-UPLC-MALS of PPI-8, PPI-13 & PPI-17. MALS results confirmed the absence of oligomers for PPI-8, PPI-13 and PPI-17.

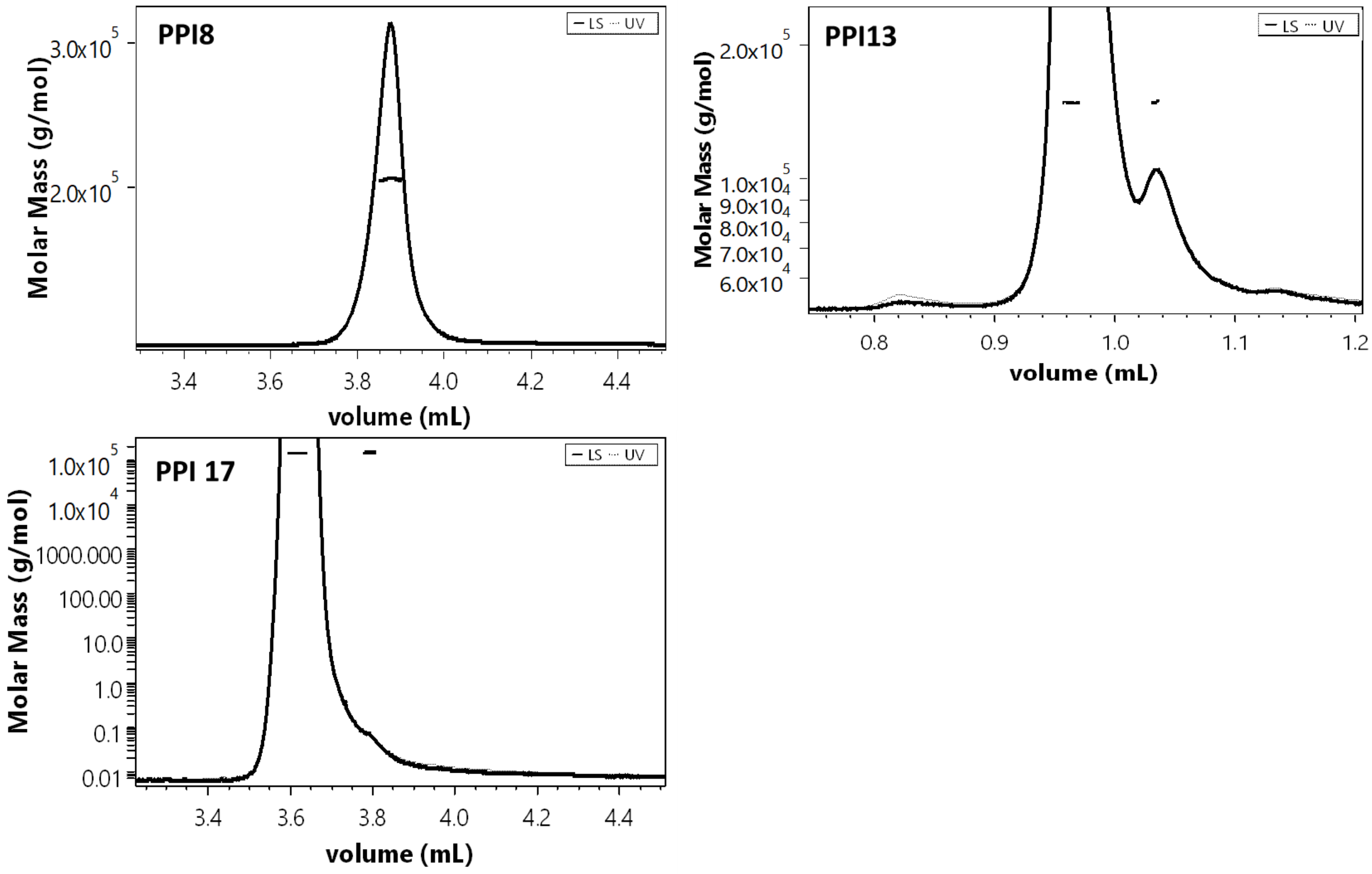

SI 2. RP-UPLC-MALS of PPI-18**.** MALS results confirmed the presence of oligomers for PPI18.

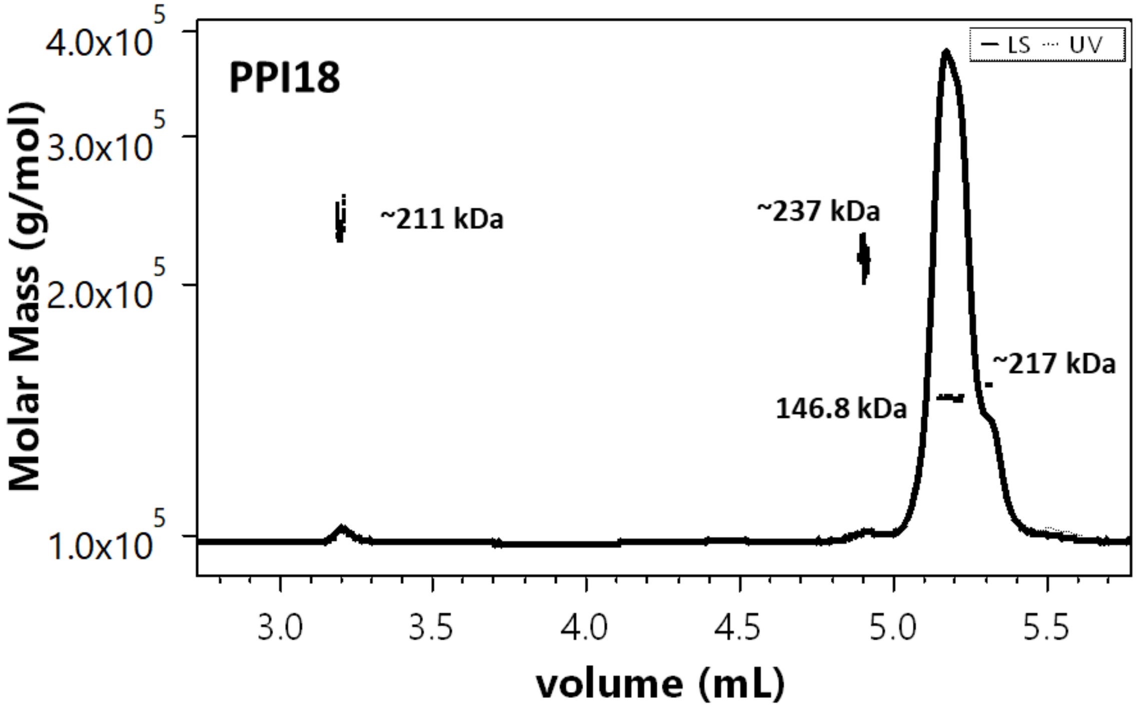

SI 3. SEC-MALS of PPI-1 Fc and Fab fragments. PPI1 Fc fragment and PPI1 Fc fragment are plotted in blue, and red lines, respectively.

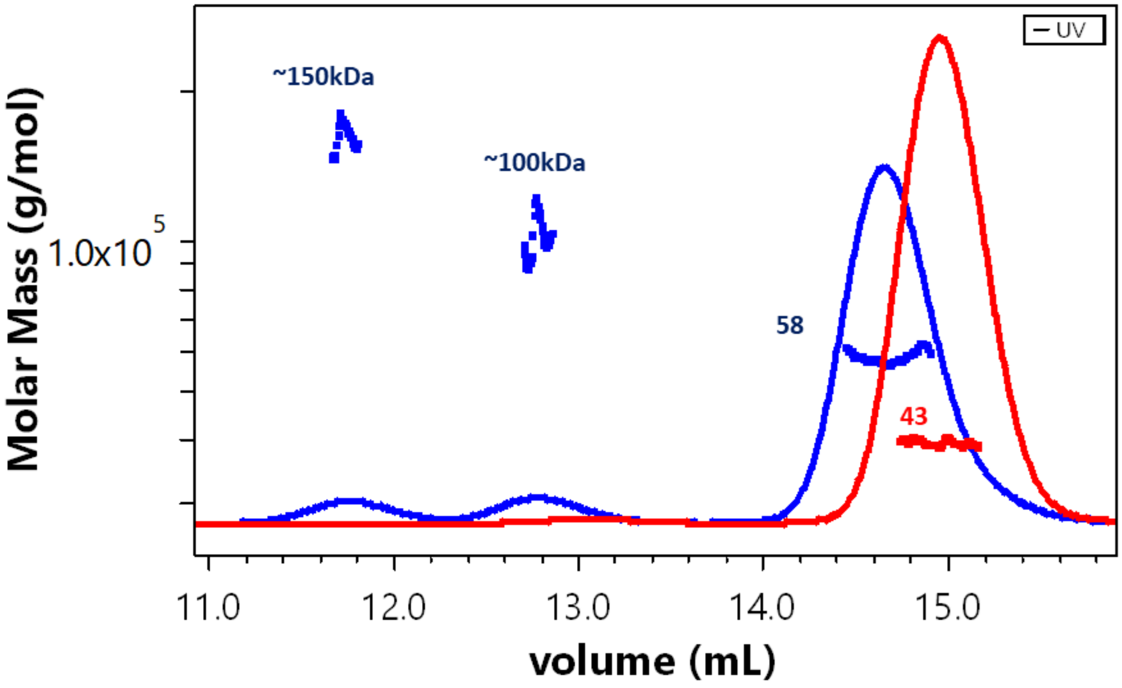

SI 4. Long-term formulations list. Formulations list for long term stability studies

Formulation	Buffer	pH	Excipient	Salt
A	10 mM Histidine	5	-	-
B	10 mM Acetate	5	-	-
C	10 mM Histidine	6.5	280 mM Proline	-
D	10 mM Histidine	6.5	140 mM ArgHCl	-
E	10 mM Histidine	6.5	280 mM Sucroce	-
F	10 mM Histidine	6.5	-	-
G	10 mM Histidine	6.5	-	140 mM NaCl
H	10 mM Phosphate	6.5	-	-

SI 5 Purity data. cIEF and SEC results of the protein bulk (i.e. before formulating).

**Method**	**cIEF**	**SEC**
Protein	Isoelectric point Ip	Monomer mass fraction (%)
PPI-01	7.2	99.7
PPI-02	9.2 – 9.3	98.3
PPI-03	9.3 – 9.4	99.8
PPI-04	8.8 – 9.0	99.1
PPI-08	9.0 – 9.2	99.7
PPI-10	8.9 – 9.2	96.3
PPI-13	8.8 – 8.9	99.4
PPI-17	8.9 – 9.1	98.5
PPI-18	5.2 – 5.6	98.3
PPI-30	6.2	100
